# Antioxidant Activity of Quercetin and Its Glucosides from Propolis: A Theoretical Study

**DOI:** 10.1038/s41598-017-08024-8

**Published:** 2017-08-08

**Authors:** Yan-Zhen Zheng, Geng Deng, Qin Liang, Da-Fu Chen, Rui Guo, Rong-Cai Lai

**Affiliations:** 1 College of Bee Science, Fujian Agriculture and Forestry University, Fuzhou, 350002 P.R. China; 2 Key Laboratory of Bioorganic Phosphorous Chemistry and Chemical Biology (Ministry of Education), Department of Chemistry, Tsinghua University, Beijing, 100084 P.R. China

## Abstract

Among the multiple components of propolis, flavonoids contribute greatly to the antioxidant activities of propolis. Flavonoids mainly exist in the form of sugar-conjugated derivatives. Quercetin glycosides represent the predominant flavonoid fraction in propolis. In this work, density functional theory (DFT) calculations were applied to analyze the antioxidative properties of quercetin and its glucosides in the gas and in the liquid phase (ethanol, water). Three main antioxidant mechanisms, hydrogen atom transfer (HAT), single electron transfer followed by proton transfer (SET-PT) and sequential proton loss electron transfer (SPLET) were used to analyze the antioxidative capacity of the investigated compounds. Solvent effects dominantly affect SET-PT and SPLET. Thus, the thermodynamically preferred mechanism can be altered. HAT and SPLET are the thermodynamically dominant mechanisms in gas and solvent phases, respectively. Therefore, in the gas phase, the sequence of the antioxidative capacity is similar with the bond dissociation enthalpy values: quercetin > quercetin-5-*O*-glucoside > quercetin-7-*O*-glucoside > quercetin-3-*O*-glucoside > quercetin-3′-*O*-glucoside > quercetin-4′-*O*-glucoside. While, in the solvent phases, the sequence is similar with the proton affinity values: quercetin-4′-*O*-glucoside > quercetin-5-*O*-glucoside > quercetin > quercetin-3-*O*-glucoside > quercetin-7-*O*-glucoside > quercetin-3′-*O*-glucoside. OH groups in B-ring and C-ring contribute mainly to the antioxidative activities of quercetin and glucosides compared with A-ring.

## Introduction

From the medical aspect, protecting cell against the damage caused by oxidation is of great importance. Free radicals and other oxidative agents have important role in the formation of many cell toxins^1^. Besides, the free radicals can induce oxidative damage in biomolecules, such as carbohydrates, proteins, lipids, and nucleic acids, which may alter the cell structure and destroy its health^2^. It has found that the oxidative damage of the cell is closely related with cardiovascular diseases, diabetes and cancer^3^.

During recent years, people has spurred greater research effort into searching functional foods that possess antioxidative activities. Propolis is a kind of resinous substance collected by honeybees from the resins of some plants such as poplars, conifers, birch, pine, alder, willow and palm^4^. Honeybees use propolis to smooth walls, seal the cracks, and keep moisture and temperature stable in the hive all year around^5^. Propolis has been widely used in folk medicine^6, 7^. Its medical efficacy mainly attribute to its broad spectrum biological properties such as anti-inflammatory, antimicrobial, antioxidant, antitumor, antiulcer and anti-HIV activities^4, 7, 8^. Among the broad biological properties of propolis, antioxidant capacity is one of the most important properties, which contributes greatly to prevent certain illnesses, including cardiovascular diseases, diabetes and cancer^9^. Among the multiple components of propolis, flavonoids contribute greatly to the antioxidant activities of propolis. According to Aljadi and Kamaruddin^10^, the antioxidant capacity of propolis is mainly due to the flavonoids they contain, and there is a high degree of correlation between flavonoids and the antioxidant capacity of propolis, although a synergic action between several compounds cannot be discounted^11^. Besides, the quantity of flavonoids is often used as a criterion to evaluate the quality of propolis^12^. In addition, it is generally believed that antioxidative effects of flavonoids represent the main reason of their intensive study.

In nature, flavonoids mainly exist in the form of sugar conjugated derivatives^13^. Isorhamnetin-3-*O*-rutinoside and rutin (quercetin-3-*O*-rhamnoglucoside) are the most common flavonoid glycosides derivatives detected from many propolis^14^. Besides these two derivatives, other flavonoid glycosides such as quercetin-3-*O*-rutinoside, quercetin-3-*O*-glucoside, kaempferol-3-*O*-rutinoside, isorhamnetin-3-*O*-rutinoside and quercetin-3-*O*-rhamnoside have also been detected from propolis^15^. Flavonoid glycosides show better solubility in water than flavonoids due to the hydrophilic nature of sugar moieties. Besides, the antioxidant activity of flavonoid may also be influenced by the glycosides^16, 17^. When compared the antioxidant activity of luteolin with its glycoside derivatives orientin, it is evident that orientin acts as a better antioxidant than luteolin from the computed values of bond dissociation enthalpy (BDE)^17^. Sugar substitution at C-8 site decreases the negative charge on the oxygen atom at C-3′ thereby results in better antioxidant potency of orientin compared to luteolin^17^.

Quercetin (2-(3,4-dihydroxyphenyl)-3,5,7-trihydroxy-4H-chromen-4-one) is a typical flavonoid ubiquitously present in different kinds of propolis^12^. It is characterized by the presence of five hydroxyl groups in positions 3, 5, 7, 3′ and 4′ of the flavonoid. Quercetin glycosides represent the predominant flavonoid fraction present in propolis and other healthy foods such as fruits and vegetables, especially onions, broccoli, apples, tea and red wine^14, 15, 18^. In this work, an attempt has been made to evaluate the antioxidative capability of quercetin and its mono-glycosides. Glucose is the most common sugar present in propolis and other foods. Therefore, glucoside was selected as the glycosides to do the detail investigation. DFT calculation has been applied to study the antioxidative properties of quercetin and its glucoside derivatives, as it is the widely and effectively used method to investigate the antioxidative properties of flavonoids and other compounds^19–29^. Water^30^ and ethanol^31^ are the common used solvent to extract flavonoids from propolis. Therefore, an attempt has also been made to investigate the antioxidant properties in these two phases and gas phase.

According from the literature, there are multiple mechanisms that related the antioxidant progress of flavonoids. In this work, flavonoids (ArOH) scavenge free radical (R^**·**^) mainly through three main antioxidant mechanisms^32–34^, namely, HAT (Eq. (1)), SET-PT (Eqs (2, 3)) and SPLET (Eqs (4–6)).

1 $${{\rm{R}}}^{\cdot }+{\rm{ArOH}}\to {\rm{RH}}+{{\rm{ArO}}}^{\cdot }$$

2 $${{\rm{R}}}^{\cdot }+{\rm{ArOH}}\to {{\rm{R}}}^{-}+{{\rm{ArOH}}}^{+\cdot }$$

3 $${{\rm{R}}}^{-}+{{\rm{ArOH}}}^{+\cdot }\to {\rm{RH}}+{{\rm{ArO}}}^{\cdot }$$

4 $${\rm{ArOH}}\to {{\rm{ArO}}}^{-}+{{\rm{H}}}^{+}$$

5 $${{\rm{ArO}}}^{-}+{{\rm{R}}}^{\cdot }\to {{\rm{ArO}}}^{\cdot }+{{\rm{R}}}^{-}$$

6 $${{\rm{R}}}^{-}+{{\rm{H}}}^{+}\to {\rm{RH}}$$

All of these mechanisms may occur in parallel, but with different rates. The thermochemistry of the reactions may be a competitive antioxidant process as the reactivity is driven by kinetics. In this work, we mainly focused on the thermochemistry of the reactions as most of the works that concern the antioxidant activity of the flavonoids have done^19–29^.

## Results and Discussion

### Molecular descriptors

**Figure 1:**
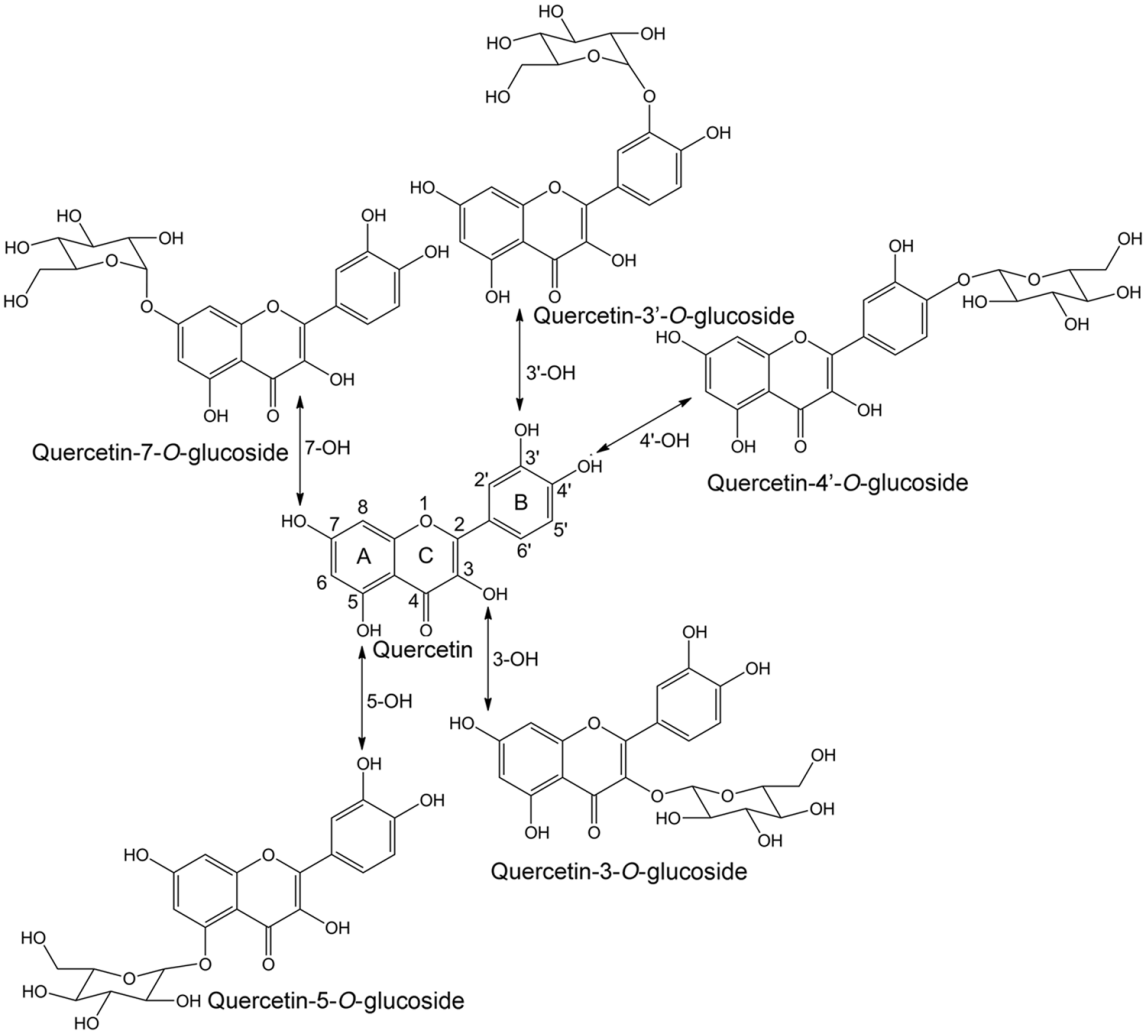
The chemical structure and atom numbering for quercetin and its glucosides.

The structure of quercetin and its different glucosides are present in Fig. 1. To characterize the antioxidant property of a flavonoid, it is vital to analyze electronegativity, electron affinity, hardness and electrophilicity index. The chemical hardness is a measure of resistance to charge transfer, while the electronegativity is a measure of the tendency to attract electrons in a chemical bond and is defined as the negative of the chemical potential in DFT^35^. The maximum electron flow between a donor and an acceptor is governed by the decomposition of binding energy between the atoms and it is determined by the factor electrophilicity index^36^. The above molecular descriptor values obtained from the total energy method for the investigated compound are displayed in Table 1. The calculated molecular properties clearly confirms that quercetin and its glucosides prefer to act as electron donor rather than electron acceptor in the studied environments^16, 17^. This is also an indication of their antioxidant activity.

**Table 1:** Molecular descriptors calculated from the total energy method obtained at M062X/6–311 + G** level of theory.

molecular descriptors	gas	ethanol	water	gas	ethanol	water
	quercetin			quercetin-3-*O*-glucoside		
electronegativity	4.36	3.94	3.94	4.69	4.07	4.08
hardness	3.57	1.86	1.79	3.59	1.93	1.86
softness	0.14	0.27	0.28	0.14	0.26	0.27
electrophilic index	2.67	4.19	4.38	3.06	4.30	4.47
	quercetin-3′-*O*-glucoside			quercetin-5-*O*-glucoside		
electronegativity	4.48	3.99	4.00	4.49	3.95	3.97
hardness	3.52	1.89	1.82	3.49	1.84	1.78
softness	0.14	0.27	0.27	0.14	0.27	0.28
electrophilic index	2.86	4.23	4.40	2.88	4.24	4.43
	quercetin-4′-*O*-glucoside			quercetin-7-*O*-glucoside		
electronegativity	4.64	4.09	4.10	4.46	3.98	3.99
hardness	3.56	1.92	1.85	3.50	1.84	1.77
softness	0.14	0.26	0.27	0.14	0.27	0.28
electrophilic index	3.03	4.37	4.54	2.84	4.30	4.49

### HAT mechanism

HAT is a major mechanism in which H atom directly transfers from antioxidant by homolytic O–H bond cleavage to free radical to break oxidative chain reaction. BDE is the numerical parameter related to HAT mechanism and characterizes the stability of the corresponding hydroxyl group. The lower BDE value indicates that the stability of the corresponding O–H bond is lower and the corresponding O–H bond can be easily broken^20, 26, 28, 29^. Hence, higher is the antioxidant capacity of the compound. In the gas and solvent phases, the calculated BDE values of quercetin and its glucosides are listed in Table 2. For quercetin, the BDE values of 4′–OH and 3–OH are lower than that of other hydroxyl groups in the gas and solvent phases, respectively. For quercetin-3′-*O*-glucoside and quercetin-4′-*O*-glucoside, the lowest BDEs are in the C-ring. While, in the remaining glucosides, the lowest BDEs is in the B-ring. For quercetin and its glucosides, except compound quercetin-5-*O*-glucoside, in which the hydrogen atom of 5–OH group is substituted by a glucose, the BDE values of 5–OH are remarkably larger than the BDE values of other OH group, indicating that H-atom subtraction from 5–OH is more difficult than other OH groups. This result can be attribute to the intramolecular hydrogen-bond between 5–OH and the nearby C=O group. In such case, hydrogen atom subtracting from 5–OH also requires breaking the intramolecular hydrogen-bond. As can be concluded from the above discussions, OH groups in B-ring and C-ring contribute mainly to the antioxidative activities of quercetin and its glucosides, whereas OH groups in A-ring play relatively little role in HAT mechanism. From the magnitude of the lowest BDE value in quercetin and different derivatives, it seems that theantioxidant activity in the gas phase can be ranged in the following sequence: quercetin > quercetin-5-*O*-glucoside > quercetin-7-*O*-glucoside > quercetin-3-*O*-glucoside > quercetin-3′-*O*-glucoside > quercetin-4′-*O*-glucoside in HAT mechanism. While in the ethanol and water phases, the sequence is: quercetin > quercetin-3′-*O*-glucoside > quercetin-7-*O*-glucoside > quercetin-5-*O*-glucoside > quercetin-4′-*O*-glucoside > quercetin-3-*O*-glucoside. Therefore, substituted the hydroxyl group by a glucose would reduce the antioxidant activity of quercetin through HAT mechanism.

**Table 2:** O–H bond dissociation enthalpies (BDE) in kJ/mol obtained at M062X/6–311 + G** level of theory.

bond	BDE			bond	BDE		
	gas	ethanol	water		gas	ethanol	water
	quercetin				quercetin-3-*O*-glucoside		
3′‒OH	369.8	360.7	350.6	3′‒OH	369.7	359.6	349.8
4′‒OH	**326.1**	344.2	336.0	4′‒OH	**333.1**	**349.4**	**341.2**
3‒OH	355.9	**343.2**	**333.1**	5‒OH	427.9	402.7	390.4
5‒OH	422.1	396.5	384.7	7‒OH	391.9	400.6	391.4
7‒OH	387.8	395.1	385.6				
	quercetin-3′-*O*-glucoside				quercetin-5-*O*-glucoside		
4′‒OH	364.1	363.2	355.7	3′‒OH	370.1	360.3	350.4
3‒OH	**356.4**	**343.5**	**333.3**	4′‒OH	**326.6**	**343.7**	**335.7**
5‒OH	423.6	397.4	385.3	3‒OH	360.3	346.9	336.7
7‒OH	387.3	395.7	386.3	7‒OH	385.7	394.7	385.5
	quercetin-4′-*O*-glucoside				quercetin-7-*O*-glucoside		
3′‒OH	372.0	364.2	354.3	3′‒OH	370.9	360.5	350.5
3‒OH	**360.8**	**347.7**	**337.2**	4′‒OH	**326.8**	**343.6**	**334.0**
5‒OH	422.2	396.0	384.0	3‒OH	356.5	344.3	335.4
7‒OH	389.1	396.6	387.1	5‒OH	421.2	396.7	385.0

**Figure 2:**
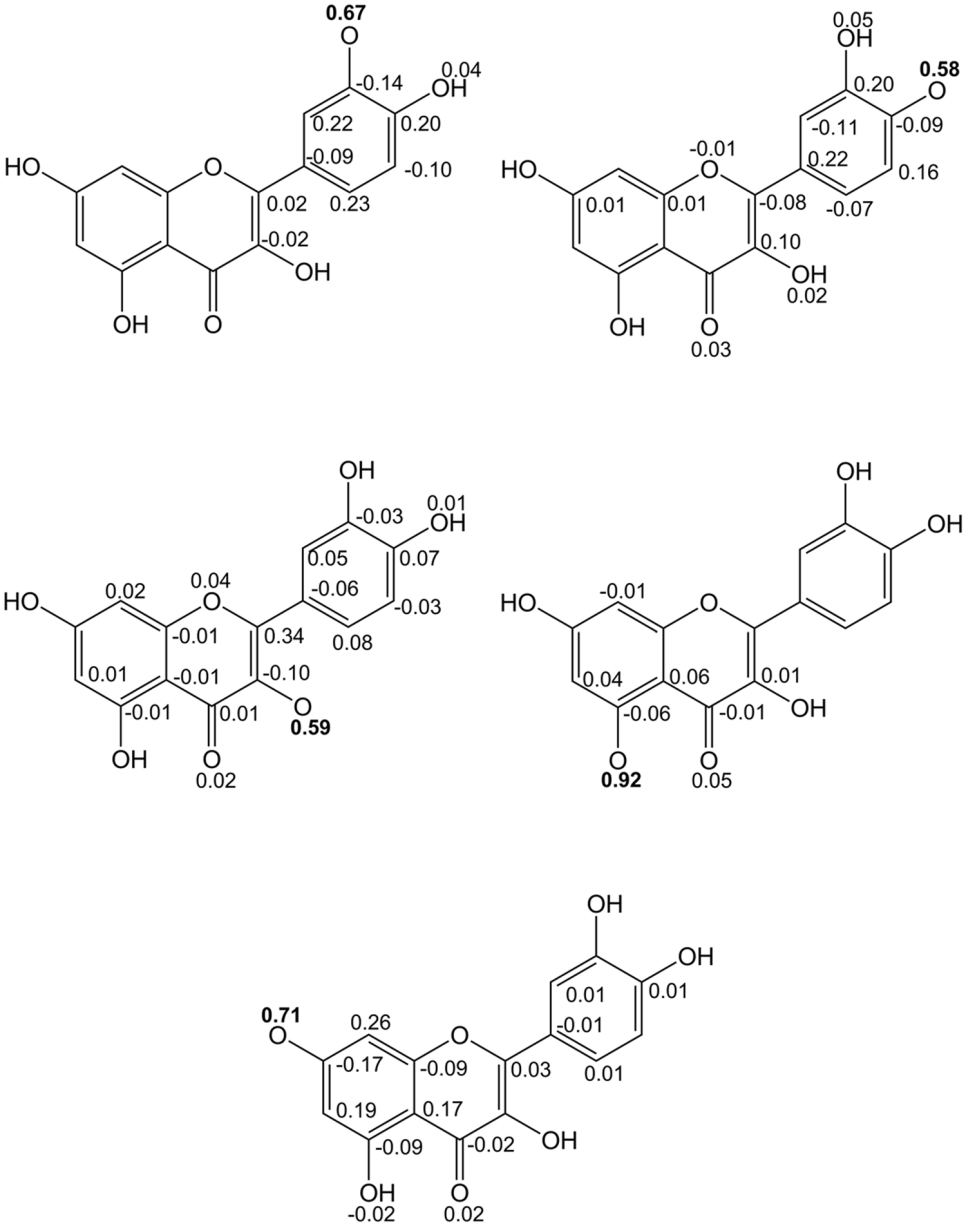
Spin density distribution of quercetin radical computed at the M062X/6–311 + G** level of theory in the gas phase.

In addition to the magnitude of BDEs, the stability of radical is another important factor that influences antioxidative activity of flavonoids. The more stable radical may indicate stronger antioxidative capacities of the compound. Spin density is a reliable parameter in rationalizing the stability of radical species^20, 28, 29^. The spin density distributions of flavonoid radicals in the gas phase were calculated and presented in Fig. 2 and Supplementary Information to explain the differences in BDE and the reactivity of the OH sites. Take the spin density of quercetin as the example. In Fig. 2, the spin density of the C5–O5^**·**^ and C7–O7^**·**^ flavonoid radicals is high on the O-atom compared to the C4′–O4′^**·**^, C3′–O3′^**·**^ and C3–O3^**·**^ radicals in the gas phase. This means that the formation of C3′–O3′^**·**^, C4′–O4′^**·**^ and C3–O3^**·**^ radicals are more favorable for spin density localization than the formation of C5–O5^**·**^ and C7–O7^**·**^ flavonoid radicals^20, 28, 29^. This also means that the stabilization of the C3′–O3′^**·**^, C4′–O4′^**·**^ and C3–O3^**·**^ radicals are higher than C5–O5^**·**^ and C7–O7^**·**^ flavonoid radicals^20, 28, 29^. The similar results can be found for the quercetin derivatives in Figures S1–S5 in the Supplementary Information. Therefore, the BDE values are lower in the B and C ring than in the A ring.

Water and ethanol are the common used solvents to extract flavonoids from propolis. When the surrounding environments of quercetin and its glucoside change from gas phase to solvent phases, the BDE values decrease. For example, the deviation between gas phase and water of BDE values is 19.2, 17.7, 19.9, 19.7, 20.4 for the 3′–OH group in quercetin, quercetin-4′-*O*-glucoside, quercetin-3-*O*-glucoside, quercetin-5-*O*-glucoside and quercetin-7-*O*-glucoside, respectively. This means that polar phase facilitates the HAT mechanism.

### SET-PT mechanism

Scavenging the free radicals may also be achieved by donating a single electron from natural flavonoid. The electron donating ability of a flavonoid is related to an extended electronic delocalization over the entire molecule. A flavonoid having a high degree of π-delocalization is more active^20, 26, 28, 29^. The calculated ionization potentials (IPs) for quercetin and its derivatives in the gas phase as well as in solvent are given in Table 3. The trend of the computed IPs for the investigated compounds is different from that of the BDEs. It is obvious that quercetin seems to donate an electron most easily, followed by quercetin-7-*O*-glucoside, quercetin-5-*O*-glucoside, quercetin-3′-*O*-glucoside, quercetin-4′-*O*-glucoside and quercetin-3-*O*-glucoside.

**Table 3:** Ionization potentials (IP) in kJ/mol obtained at M062X/6–311 + G** level of theory.

molecule	IP		
	gas	ethanol	water
quercetin	**733.3**	**536.1**	**534.6**
quercetin-3′-*O*-glucoside	738.4	537.6	536.2
quercetin-4′-*O*-glucoside	744.7	546.8	546.6
quercetin-3-*O*-glucoside	770.0	556.7	554.2
quercetin-5-*O*-glucoside	738.2	537.3	535.4
quercetin-7-*O*-glucoside	737.2	537.0	534.7

Proton dissociation enthalpy (PDE) characterizes the second step of SET–PT mechanism and can indicate the thermodynamically preferred OH group for deprotonation from the cation radical. The calculated PDEs for quercetin and its glucosides in the gas phase as well as in solvents are given in Table 4. For quercetin, the PDE values of 4′–OH and 3–OH are lower than that of other hydroxyl groups in the gas and solvent phases, respectively. In the studied environments, the lowest PDEs in quercetin-3′-*O*-glucoside and quercetin-4′-*O*-glucoside are in the C-ring. While, in the remaining glucosides, the lowest PDEs is in the B-ring. The trends observed for PDEs are analogous with those for BDEs, because the second step of the SET–PT mechanism also results in the quercetin radical formation. The PDE values of 5–OH and 7–OH in A-ring are remarkably larger than that of other OH groups in B-ring and C-ring. In such case, hydrogen atom abstraction from A-ring is more difficult than B-ring and C-ring. In other words, OH groups in B-ring and C-ring contribute greatly to the second step of SET-PT mechanism, whereas OH groups in A-ring play relatively little role.

**Table 4:** Proton dissociation enthalpies (PDE) in kJ/mol obtained at M062X/6–311 + G** level of theory.

bond	PDE			bond	PDE		
	gas	ethanol	water		gas	ethanol	water
	quercetin				quercetin-3-*O*-glucoside		
3′‒OH	947.3	6.7	15.2	3′‒OH	915.6	‒14.0	‒5.1
4′‒OH	**903.5**	−9.8	0.6	4′‒OH	**879.0**	**−24.3**	**−13.7**
3‒OH	933.4	**−10.9**	**−2.3**	5‒OH	973.7	29.0	35.5
5‒OH	999.5	42.5	49.3	7‒OH	937.8	26.9	36.5
7‒OH	965.2	41.1	50.2				
	quercetin-3′-*O*-glucoside				quercetin-5-*O*-glucoside		
4′‒OH	946.7	8.9	18.8	3′‒OH	948.8	7.2	15.1
3‒OH	**939.0**	**−11.1**	**−3.7**	4′‒OH	**905.3**	**−9.5**	**0.4**
5‒OH	1006.2	43.1	48.4	3‒OH	939.0	−6.1	1.4
7‒OH	969.9	41.4	49.3	7‒OH	964.4	41.7	50.3
	quercetin-4′-*O*-glucoside				quercetin-7-*O*-glucoside		
3′‒OH	943.2	0.4	7.0	3′‒OH	948.6	5.9	14.4
3‒OH	**932.0**	**−16.1**	**−10.1**	4′‒OH	**904.5**	**−10.5**	**−2.1**
5‒OH	993.5	32.2	36.7	3‒OH	934.2	−10.3	−0.7
7‒OH	960.4	32.8	39.8	5‒OH	998.9	42.1	48.9

It is well known that the charged species are quite sensitive to the polarity of the environments^26, 28, 29^. The SET–PT mechanism is related with the formation and breaking of flavonoid cation radical which possess a positive charge. As expected, the values of IPs and PDEs in solvent phases would significantly change compared with in the gas phase. As can be seen from Tables 3 and 4, the IP and PDE values in ethanol and water are dramatically lower than that in the gas phase. The average deviation between gas phase and solvent environments were more than 200 kJ/mol and 900 kJ/mol for IP and PDE, respectively. This confirms that the polar medium can facilitate electron donation. The causes may be that: (1) the radical cation is more stable in the polar environments; (2) the delocalization and conjugation of the π-electrons of the quercetin and its glucoside derivatives molecule is more delocalized in polar environments^37^.

For multiple steps mechanism, the first step is the most important one from the thermodynamic point. Therefore, for SET-PT mechanism, from the sequence of IP values, the antioxidant activity of the investigated compounds can be ranged in the following order: quercetin > quercetin-7-*O*-glucoside > quercetin-5-*O*-glucoside > quercetin-3′-*O*-glucoside > quercetin-4′-*O*-glucoside > quercetin-3-*O*-glucoside in the studied environments. Therefore, substituted the hydroxyl group by a glucose would reduce the antioxidant activity of quercetin through SET-PT mechanism.

### SPLET mechanism

SPLET is the third important antioxidative mechanism by which antioxidant trap free radicals. Previous studies for phenolic antioxidants have confirmed the important role of SPLET in polar solvents^26, 28, 29^. Thus, the possible contribution of SPLET was investigated to explore the radical scavenging activity of quercetin and its derivatives.

According to the mechanism of SPLET, formation of flavonoid anion is the first step, which is governed by the acid strength of OH group in flavonoid. It is especially important not only in the first step of SPLET mechanism but also connected with metal chelation action of flavonoids. Table 5 presents the calculated proton affinities (PAs) values in gas phase as well as in solvents for quercetin and its glucosides. By comparison, it can be deduced that the PA values of 3′–OH is smaller than that of other hydroxyl groups for quercetin-4′-*O*-glucoside in all the studied environments. For quercetin and other glucosides, the PA values of 4′–OH are always smaller than that of other hydroxyl groups in all the studied environments. This clearly shows that formation of flavonoid –O^−^ anion in B-ring is easier than that of other anions. It can be also seen that the PA values of 5–OH are always larger than that of other hydroxyl groups in quercetin and its different derivatives. This demonstrates that formation of 5–O^−^ anion is more difficult than other anions. This can be ascribed to the formation of intramolecular hydrogen-bond between 5–OH and adjacent carbonyl group. Therefore, for quercetin and its glucosides, the lowest PA value can be ascribed predominantly to B-ring, while, the largest PA value is mainly on the A-ring.

**Table 5:** Proton affinities (PA) in kJ/mol obtained at M062X/6–311 + G** level of theory.

bond	PA			bond	PA		
	gas	ethanol	water		gas	ethanol	water
	quercetin				quercetin-3-*O*-glucoside		
3′‒OH	1424.1	132.5	125.8	3′‒OH	1408.7	135.5	128.6
4′‒OH	**1338.8**	**107.6**	**105.5**	4′‒OH	**1314.6**	**107.7**	**106.0**
3‒OH	1402.9	122.8	116.1	5‒OH	1404.0	135.7	128.7
5‒OH	1425.1	130.9	123.5	7‒OH	1342.7	111.3	108.2
7‒OH	1364.5	110.5	106.8				
	quercetin-3′-*O*-glucoside				quercetin-5-*O*-glucoside		
4′‒OH	**1344.5**	**109.8**	**107.4**	3′‒OH	1415.9	134.3	127.2
3‒OH	1386.4	125.2	119.9	4′‒OH	**1329.6**	**107.1**	**104.1**
5‒OH	1411.3	131.4	124.6	3‒OH	1396.5	131.0	123.8
7‒OH	1348.7	120.4	118.9	7‒OH	1334.9	110.4	107.9
	quercetin-4′-*O*-glucoside				quercetin-7-*O*-glucoside		
3′‒OH	**1348.8**	**100.5**	**96.8**	3′‒OH	1421.2	134.2	127.6
3‒OH	1379.8	120.2	114.7	4′‒OH	**1332.5**	**108.8**	**107.0**
5‒OH	1409.8	130.5	123.8	3‒OH	1391.6	123.6	117.8
7‒OH	1351.1	110.0	107.2	5‒OH	1404.9	132.4	126.1

The first step of SPLET mechanism is also related with the formation charge species. Due to the high solvation enthalpies of proton and anion, the PA values decrease drastically from gas phase to solution. This result is similar to the case of PDE. The average deviations of PAs between the gas phase and the solvents were exceeding 1200 kJ/mol. This indicates that polar solvents can accelerate the deprotonation process of flavonoid.

From the magnitude of the lowest PA values in quercetin and different glucosides, it seems that in the gas phase, proton is most easily heterolytic cleavage from quercetin-3-*O*-glucoside, followed with quercetin-5-*O*-glucoside, quercetin-7-*O*-glucoside, quercetin, quercetin-3′-*O*-glucoside and quercetin-4′-*O*-glucoside. In the ethanol and water phases, proton is most easily heterolytic cleavage from quercetin-4′-*O*-glucoside, followed with quercetin-5-*O*-glucoside, quercetin, quercetin-3-*O*-glucoside, quercetin-7-*O*-glucoside, quercetin-3′-*O*-glucoside.

According to the mechanism of SPLET, formation of flavonoid radical is the second step, which is governed by electron transfer enthalpy (ETE). Table 6 presents the calculated ETE values in gas phase as well as in solvents for quercetin and its derivatives. In the gas phase, the lowest ETEs were found for 3′–O^**·**^ radical formation in quercetin, quercetin-3-*O*-glucoside, quercetin-5-*O*-glucoside and quercetin-7-*O*-glucoside. In quercetin-3′-*O*-glucoside and quercetin-4′-*O*-glucoside, lowest ETEs were found for 3–O^**·**^ formation. In the ethanol and water phases, lowest ETE was found for 3′–O^**·**^ formation in quercetin-3-*O*-glucoside. While, the lowest ETEs were found for 3–O^**·**^ radical formation in quercetin and other derivatives. Although, the lowest ETEs cannot be ascribed predominantly to B ring or C ring, the biggest ETE values can be found in 7–O^**·**^ radical formation. As can be concluded from the above discussions, OH groups in B-ring and C-ring contribute greatly to the second step of SPLET mechanism, whereas OH groups in A-ring play relatively little role.

**Table 6:** Electron transfer enthalpies (ETE) in kJ/mol obtained at M062X/6–311 + G** level of theory.

bond	ETE			bond	ETE		
	gas	ethanol	water		gas	ethanol	water
	quercetin				quercetin-3-*O*-glucoside		
3′‒OH	**261.6**	411.3	424.1	3′‒OH	**276.9**	**407.1**	**420.5**
4′‒OH	303.2	419.6	429.8	4′‒OH	334.4	424.7	434.5
3‒OH	268.9	**403.7**	**416.3**	5‒OH	339.8	450.0	461.0
5‒OH	312.8	448.6	460.4	7‒OH	365.1	472.3	482.4
7‒OH	339.2	467.6	478.1				
	quercetin-3′-*O*-glucoside				quercetin-5-*O*-glucoside		
4′‒OH	335.5	425.9	436.1	3′‒OH	**270.1**	409.0	422.5
3‒OH	**285.9**	**401.0**	**412.6**	4′‒OH	312.9	416.2	427.0
5‒OH	328.2	449.0	460.0	3‒OH	279.7	**399.0**	**412.1**
7‒OH	354.6	468.9	478.2	7‒OH	366.6	470.7	480.7
	quercetin-4′-*O*-glucoside				quercetin-7-*O*-glucoside		
3′‒OH	336.8	446.7	456.7	3′‒OH	**265.6**	409.3	422.2
3‒OH	**296.9**	**410.5**	**421.8**	4′‒OH	310.2	417.9	427.7
5‒OH	328.3	448.5	459.4	3‒OH	280.8	**403.7**	**415.6**
7‒OH	356.2	469.6	479.2	5‒OH	332.2	447.4	458.1

In solution, ETEs are higher than the corresponding gas phase values. The average deviations between the gas phase and ethanol are exceeding 90 kJ/mol, and that between the gas phase and water are more than 110 kJ/mol. These results indicate that the polar environments is less favored in the second step of SPLET mechanism.

In SPLET mechanism, according to the value of PAs, the antioxidant activity of the investigated compounds can be ranged in the following order: > quercetin-3-*O*-glucoside > quercetin-5-*O*-glucoside > quercetin-7-*O*-glucoside > quercetin > quercetin-3′-*O*-glucoside > quercetin-4′-*O*-glucoside in the gas phase. Therefore, substituted the hydroxyl group by a glucose in the 3-site, 5-site and 7-site would increase the antioxidant activity of quercetin through SPLET mechanism in the gas phase. While, substituted the other hydroxyl group by a glucose would reduce the antioxidant activity of quercetin through SPLET mechanism. In the solvent phases, the sequence is: quercetin-4′-*O*-glucoside > quercetin-5-*O*-glucoside > quercetin > quercetin-3-*O*-glucoside > quercetin-7-*O*-glucoside > quercetin-3′-*O*-glucoside. Therefore, substituted the hydroxyl group by a glucose in the 4′-site and 5-site would increase the antioxidant activity of quercetin through SPLET mechanism in the solvent phases. While, substituted the other hydroxyl group by a glucose would reduce the antioxidant activity of quercetin through SPLET mechanism.

### Frontier orbitals

**Figure 3:**
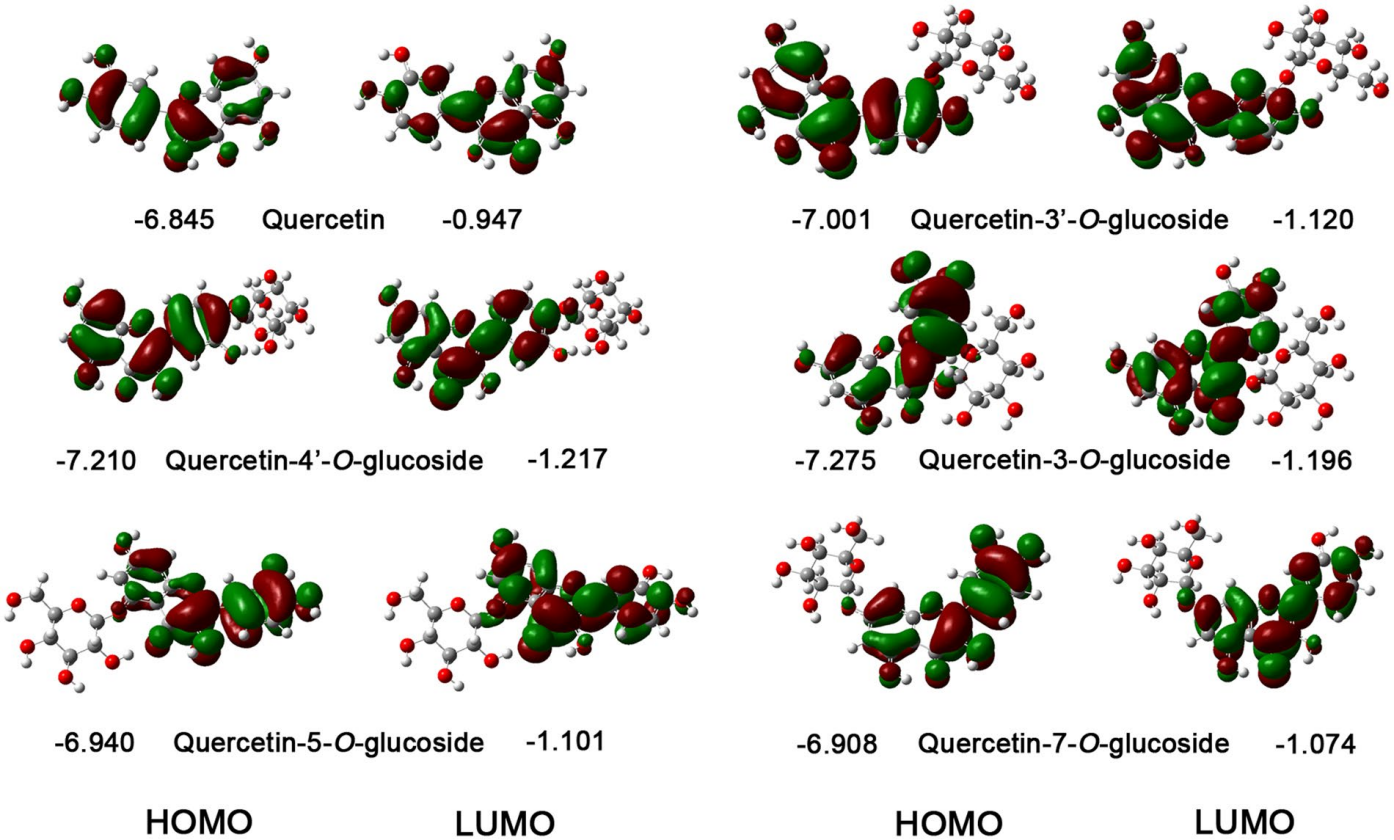
The energy and distribution of HOMO and LUMO for quercetin and its glucosides in the gas phase.

The energy and distribution of the frontier orbitals are also important parameters that correlate with the antioxidative activity of the flavonoids. The calculated frontier orbital distributions and energies in the gas phase for quercetin and its glucosides are present in Fig. 3. From Fig. 3, it can be obtained that similar distributions were present in the HOMO and LUMO orbitals for the investigated compounds. More importantly, the HOMO orbitals are mainly localized on the B-ring and C-ring, whereas the LUMO orbitals are distributed over the whole molecule. This means that the OH groups in the B-ring and C ring in quercetin and its glucosides would be more easily attacked by free radicals^20, 28, 29^. This result is the same with the BDE and PA values which govern the HAT and SPLET mechanisms, respectively.

The electron donating ability of flavonoid is related with the HOMO energies. The molecules with higher HOMO orbital energy have stronger electron donating abilities^20, 28, 29^. It can be observed from Fig. 3 that quercetin provided the highest HOMO energy (−6.845 eV), followed by quercetin-7-*O*-glucoside (−6.908 eV), quercetin-5-*O*-glucoside (−6.940 eV), quercetin-3′-*O*-glucoside (−7.001 eV), quercetin-4′-*O*-glucoside (−7.210 eV) and quercetin-3-*O*-glucoside (−7.275 eV). This clearly shows that quercetin has the strongest electron-donating capability among the studied compounds. By comparison, the predicted electron-donating capability sequence according to HOMO energies was found to be the same as that for the IP.

### Thermodynamically preferred mechanism

Strictly speaking, thermodynamically favored mechanism is decided by free energy (Δ_r_ *G*). The calculated equation for free energy is Δ_r_ *G* = Δ_r_ *H* − *T*Δ_r_ *S*. According from the equation, Δ_r_ *G* is determined by Δ_r_ *H* and *T*Δ_r_ *S*. However, in the case of studied reactions, the absolute values of the entropic term, *T*Δ_r_ *S*, reach only few units or tens of kJ/mol^26, 38^. Therefore, the free energies are mainly influenced by the enthalpy term Δ_r_ *H*. As stated in the experimental section, IPs and PAs are reaction enthalpies related to the first step of the SET–PT and SPLET mechanisms, and therefore the HAT, SET-PT and SPLET mechanisms are mainly governed by BDEs, IPs and PAs, respectively. Thus, the thermodynamically preferred reaction pathway involved in the free radical scavenging process can be determined by the BDEs, IPs and PAs. By comparison, it is found that in the gas phase, the calculated IPs and PAs of quercetin and its glucosides are significantly higher than the BDEs, and hence from the thermodynamic point of view, HAT represents the most favorable process in the gas phase. From the magnitude of the lowest BDE value, in the gas phase, the antioxidative capacity increase in the order: quercetin-4′-*O*-glucoside < quercetin-3′-*O*-glucoside < quercetin-3-*O*-glucoside < quercetin-7-*O*-glucoside < quercetin-5-*O*-glucoside < quercetin. Therefore, substituted the hydroxyl group by a glucose would reduce the antioxidant activity of quercetin in the gas phase. In ethanol and water phases, large decrease in the PAs makes SPLET more favorable mechanism than HAT and SET–PT. Available experimental and theoretical studies for flavonoids have also confirmed the important role of the SPLET mechanism in polar solvents. From the magnitude of the lowest PA value, in the ethanol and water phases, the antioxidative capacity can be ranked in the following order: quercetin-4′-*O*-glucoside > quercetin-5-*O*-glucoside > quercetin > quercetin-3-*O*-glucoside > quercetin-7-*O*-glucoside > quercetin-3′-*O*-glucoside. Therefore, substituted the hydroxyl group by a glucose in the 4′-site and 5-site would increase the antioxidant activity of quercetin in the solvent phases. While, substituted the other hydroxyl groups by a glucose would reduce the antioxidant activity of quercetin.

In the studied environments, the calculated IPs are significantly higher than the BDEs and PAs. Therefore, SET–PT is the thermodynamically least preferred mechanism in the studied environments.

Results obtained for studied environments indicate that HAT mechanism can be assigned to the 4′–OH or 3–OH group in the ring C or ring B for quercetin and its glucosides. For SPLET, thermodynamically preferred OH groups are 3′–OH or 4′–OH in ring B. Therefore, from the thermodynamically aspect, OH groups in B-ring and C-ring contribute mainly to the antioxidative activities of quercetin and its glucosides.

## Conclusions

In this work, the antioxidative properties of quercetin and its glucosides in the gas, ethanol and water phases were studied from theoretical aspect. The calculated molecular properties (electronegativity, ionization potential, electron affinity, hardness and electrophilicity index) clearly confirm that quercetin and its glucosides prefer to act as electron donor rather than electron acceptor in the studied environments. This is an indicator of their antioxidative activity. Based on the above result, three main antioxidant mechanisms, namely HAT, SET-PT and SPLET were taken into account to analyze the antioxidative capacity of quercetin and its glucoside derivatives in the gas, ethanol and water phases.

As for the HAT mechanism, from the magnitude of the lowest BDE values, in the gas phase the sequence of antioxidant ability of the investigated compounds is: quercetin >quercetin-5-*O*-glucoside > quercetin-7-*O*-glucoside > quercetin-3-*O*-glucoside > quercetin-3′-*O*-glucoside > quercetin-4′-*O*-glucoside. While, in the solvent environments, the sequence is: quercetin > quercetin-3′-*O*-glucoside > quercetin-7-*O*-glucoside > quercetin-5-*O*-glucoside > quercetin-4′-*O*-glucoside > quercetin-3-*O*-glucoside. Hydroxyl group on the 4′-position in the B ring and 3-position in the C ring have higher H-atom donation ability than the other hydroxyl groups and strongly participate in the radical scavenging activity.

As for SET-PT mechanism, in the studied environments, the antioxidant activity of the investigated compounds can be ranged in the following order: quercetin > quercetin-7-*O*-glucoside, quercetin-5-*O*-glucoside, quercetin-3′-*O*-glucoside, quercetin-4′-*O*-glucoside and quercetin-3-*O*-glucoside.

In SPLET mechanism, in the gas phase, the antioxidant activity of the investigated compounds can be ranged in the following order: quercetin-3-*O*-glucoside > quercetin-5-*O*-glucoside > quercetin-7-*O*-glucoside > quercetin > quercetin-3′-*O*-glucoside > quercetin-4′-*O*-glucoside in the gas phase. While in the solvent phases, the sequence is: quercetin-4′-*O*-glucoside > quercetin-5-*O*-glucoside > quercetin > quercetin-3-*O*-glucoside > quercetin-7-*O*-glucoside > quercetin-3′-*O*-glucoside.

Solvents induce significant changes in the enthalpies of charged species, which dominantly affects SET-PT and SPLET, especially the value of PAs. Thus, for quercetin and its glucosides, the thermodynamically preferred mechanism of the antioxidative progress can be altered by the environments. For the antioxidative progress, HAT is the thermodynamically dominant process in gas phase, while SPLET is more favored in ethanol and water environments. Therefore, in the gas phase, the sequence of the antioxidative capacity is: quercetin > quercetin-5-*O*-glucoside > quercetin-7-*O*-glucoside > quercetin-3-*O*-glucoside > quercetin-3′-*O*-glucoside > quercetin-4′-*O*-glucoside. While, in the solvent phases, the sequence is: quercetin-4′-*O*-glucoside > quercetin-5-*O*-glucoside > quercetin > quercetin-3-*O*-glucoside > quercetin-7-*O*-glucoside > quercetin-3′-*O*-glucoside.

From the calculated results, OH groups in B-ring and C-ring contribute mainly to the antioxidative activities of quercetin and glucosides compared with A-ring.

These studies deepen our understanding on the antioxidative capacity of quercetin and its glucosides in gas, ethanol and water phases. It may also shed light on the studies of flavonoids and glucosides in the studied environments and other solvents in the future.

## Methods

### Computational details

#### Molecular descriptive parameters

The molecular descriptive parameters including the electronegativity (*χ*), IP, electron affinity (EA), chemical hardness (*η*), softness (*S*), and electrophilicity index (*ω*)^39^ are very vital to characterize the antioxidant property of flavonoids. The electronic properties are computed by calculating the total energy (*E*_V_) of the species. The IP is calculated as the energy difference between the compound derived from electron-transfer (radical cation) and the respective neutral compound: IP_E_ = *E*_cation_ − *E*_n_. The EA is computed as the energy difference between the neutral molecule and the anion molecule: EA = *E*_n_ − *E*_anion_. The *χ*, *η*, *S* and *ω* are computed by using the Eqs (7–10)^36, 39–41^.

7 $$\chi =-\mu \approx ({\rm{IP}}+{\rm{EA}})/2$$

8 $$\eta \approx ({\rm{IP}}-{\rm{EA}})/2$$

9 $$S\approx 1/(2\eta )$$

10 $$\omega \approx {\mu }^{2}/2\eta $$

#### Antioxidant mechanism

The HAT mechanism is a hydrogen atom abstraction from the flavonoid hydroxyl groups to the free radical by homolytic cleavage. The capacity of this mechanism is essentially driven by the O–H BDE. The BDE was calculated by Eq. (11). The lower BDE parameter characterizes better antioxidant property of the investigated compound.

11 $${\rm{BDE}}=H({{\rm{ArO}}}^{\cdot })+H({{\rm{H}}}^{\cdot })\,-\,H({\rm{ArOH}})$$

The SET-PT mechanism consists of two steps. The first step (electron loss or electron transfer) is followed by the formation of the ArOH^+^ cation radical. This mechanism is governed by IP. The second step is the heterolytic O–H bond dissociation, which is driven by PDE. The IP and PDE were calculated by Eqs (12) and (13), respectively. Antioxidants with lower IP values enhance the probability of radical anion generation through the direct electron transfer.

12 $${\rm{IP}}=H({{\rm{ArOH}}}^{+\cdot })+H({{\rm{e}}}^{-})\,-\,H({\rm{ArOH}})$$

13 $${\rm{PDE}}=H({{\rm{ArO}}}^{\cdot })+H({{\rm{H}}}^{+})\,-\,H({{\rm{ArOH}}}^{+\cdot })$$

In the SPLET mechanism, the first step is the proton transfer, followed by the electron transfer. The first step is driven by PA of ArOH and the second step is governed by ETE of ArO^-^. The PA and ETE were calculated by Eqs (14) and (15), respectively. Antioxidants with lower PA and ETE values are expected to have higher antioxidative activity.

14 $${\rm{PA}}=H({{\rm{ArO}}}^{-})+H({{\rm{H}}}^{+})\,-\,H({\rm{ArOH}})$$

15 $${\rm{ETE}}=H({{\rm{ArO}}}^{\cdot })+H({{\rm{e}}}^{-})\,-\,H({{\rm{ArO}}}^{-})$$

The calculated gas-phase *H*(H^+^) and *H*(e−) are 6.197 kJ/mol and 3.145 kJ/mol^42^ from literature, respectively. Hydrogen atom solvation enthalpies were taken from the work by Parker^43^ and Bizarro *et al*.^44^. Proton and electron solvation enthalpies were taken from the work by Rimarcik *et al*.^38^.

All of the calculations were carried out using the Gaussian 09 program suite^45^. The geometries and vibrational spectra analysis were obtained using M062X/6–311 + G**. The minimum of the Cartesian coordinates for each of the structures used in this study were put in Figures S6–S11 in the Supplementary Information. The absence of imaginary frequencies confirmed that the optimized structure is a local minimum. The frontier orbital distribution and energy of HOMO and LOMO orbitals as well as the spin density of the radicals were also carried out at M062X/6-311 + G** level. Single point energy calculations were performed at the M062X/6-311 + G** level of theory. The solvent effect was performed by using the Tomasi’s polarized continuum model (PCM)^46^ employed in the Gaussian 09 package.

## Electronic supplementary material

Supplementary Information
