# Supplementary material for: Antioxidant Activity of Quercetin and Its Glucosides from Propolis: A Theoretical Study
Source: Sci Rep. 2017 Aug 8;7:7543. doi: 10.1038/s41598-017-08024-8 (PMC5548903; doi:10.1038/s41598-017-08024-8)
Supplement: Supplementary file 1 — Supplementary Information [file 41598_2017_8024_MOESM1_ESM.pdf]

Supplementary Information for:

Antioxidant Activity of Quercetin and Its Glucosides from Propolis: A Theoretical Study

Yan-Zhen Zheng,[a] Geng Deng,[b] Qin Liang,[a] Da-Fu Chen[a*], Rui Guo[a] & Rong-Cai Lai[a]

[a]*College of Bee Science, Fujian Agriculture and Forestry University, Fuzhou 350002, P. R. China*

[b]*Key Laboratory of Bioorganic Phosphorous Chemistry and Chemical Biology (Ministry of Education), Department of Chemistry, Tsinghua University, Beijing 100084, P. R. China*

***To whom correspondence should be addressed.**

Dr. Da-Fu Chen

Email: dfchen826@163.com

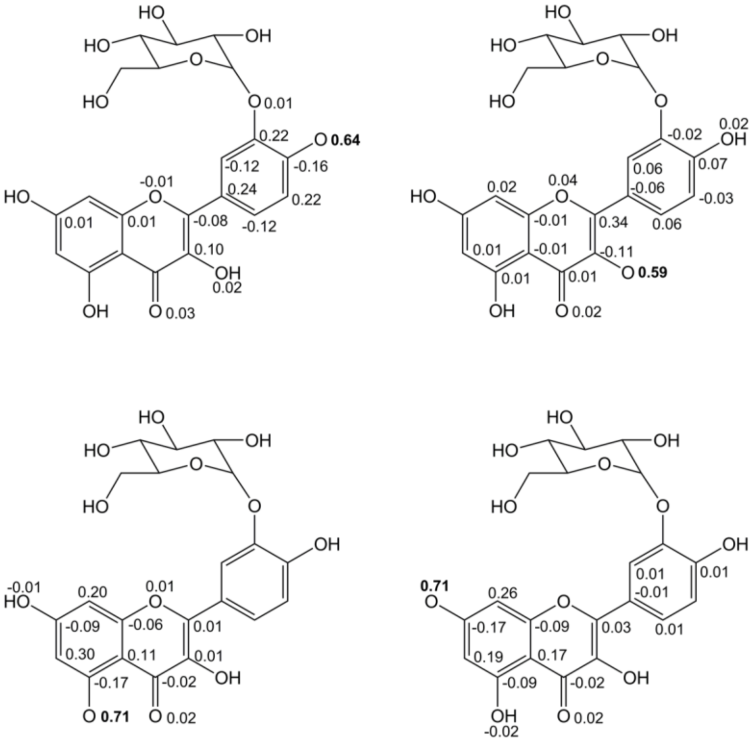

Figure S1. Spin density distribution of quercetin-3’-*O*-glucoside radical computed at the M062X/6-311+G** level of theory in the gas phase.

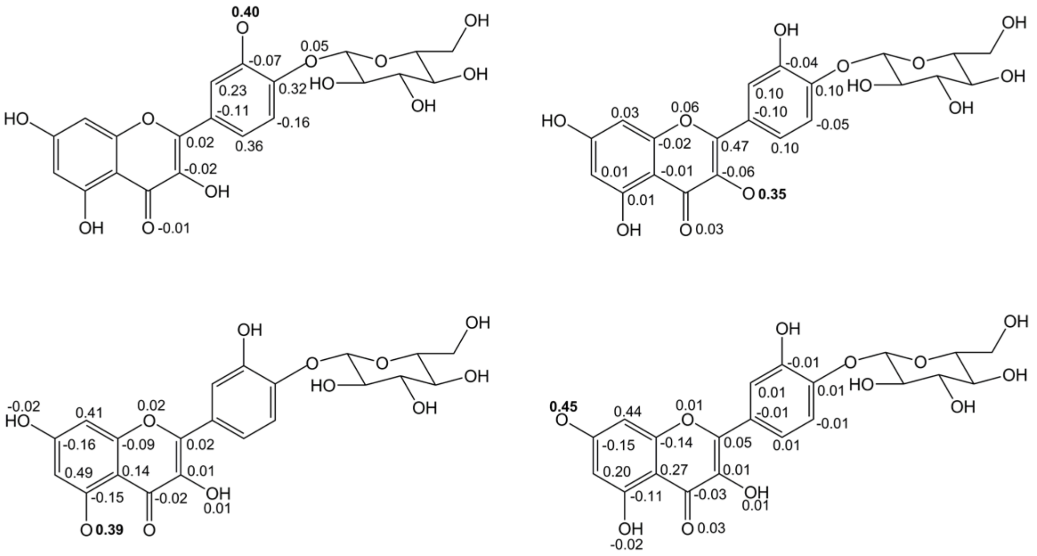

Figure S2. Spin density distribution of quercetin-4’-*O*-glucoside radical computed at the M062X/6-311+G** level of theory in the gas phase.

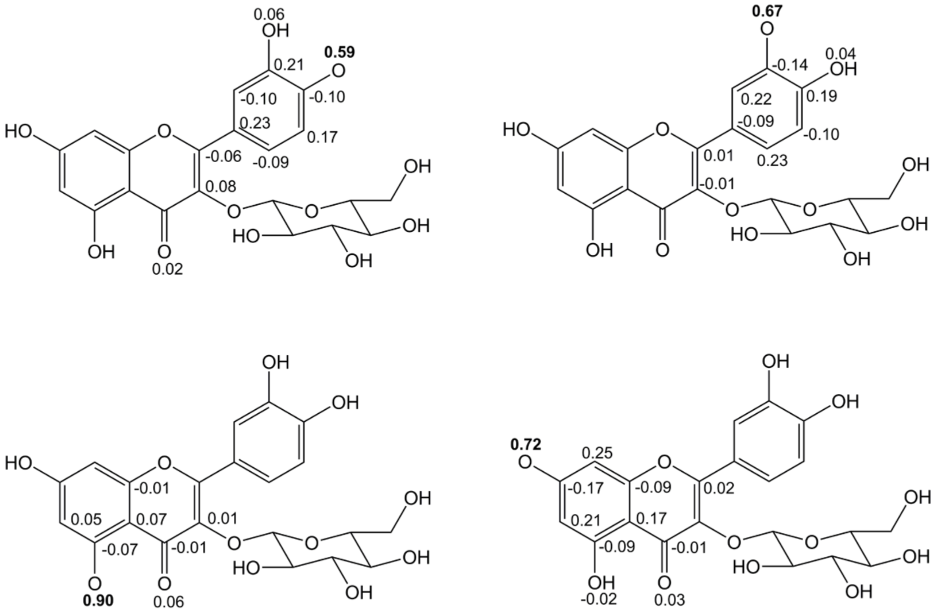

Figure S3. Spin density distribution of quercetin-3-*O*-glucoside radical computed at the M062X/6-311+G** level of theory in the gas phase.

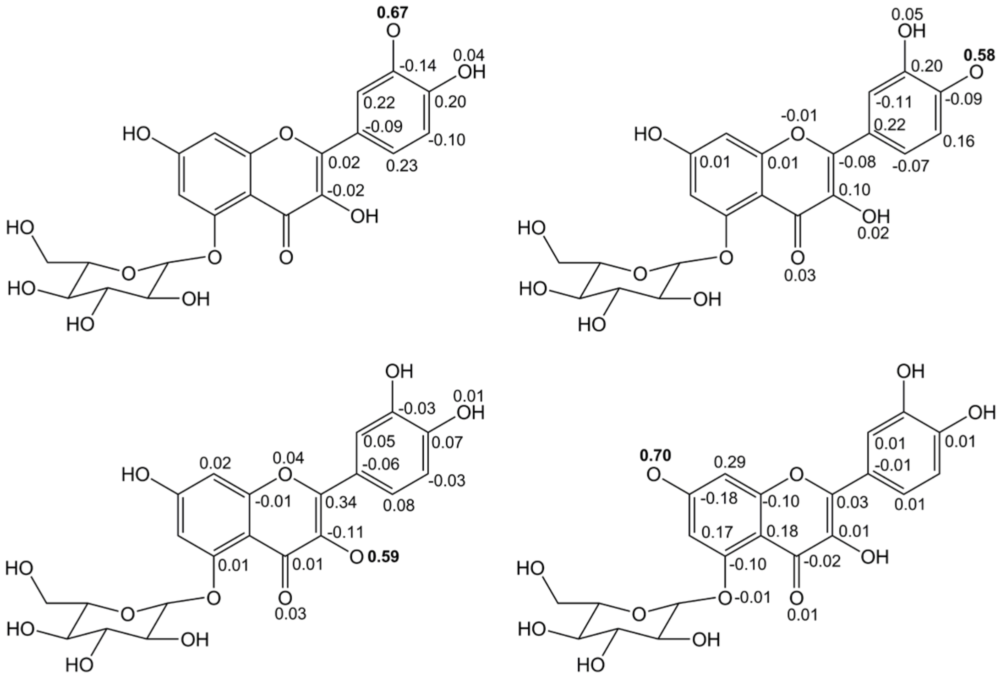

Figure S4. Spin density distribution of quercetin-5-*O*-glucoside radical computed at the M062X/6-311+G** level of theory in the gas phase.

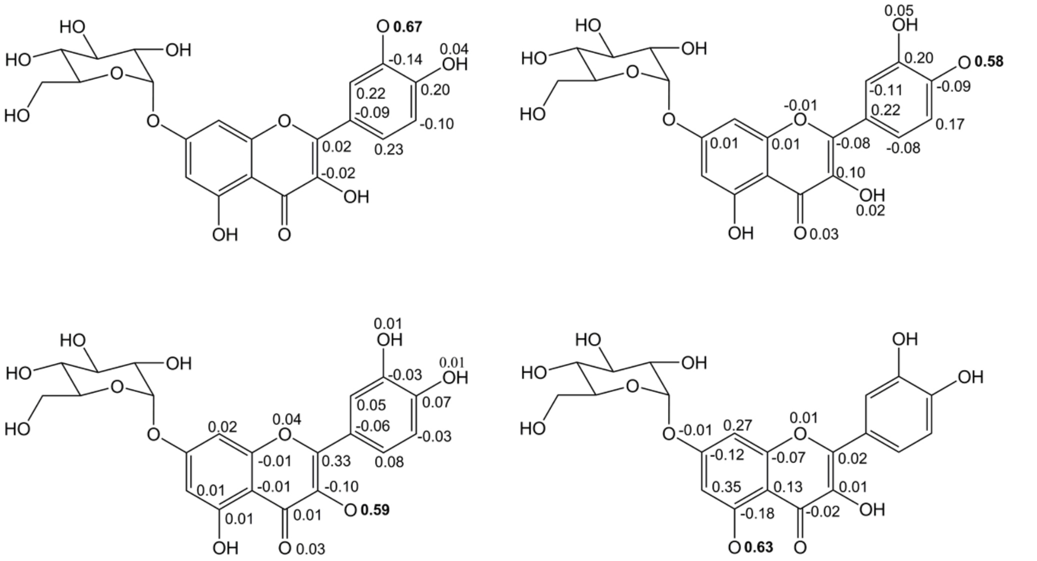

Figure S5. Spin density distribution of quercetin-7-*O*-glucoside radical computed at the M062X/6-311+G** level of theory in the gas phase.

```
C                -0.42928700   -0.52525700   -0.02249600
C                 0.24918600   -1.70219900    0.03256500
C                 1.70504500   -1.73513100    0.05315400
O                 2.27601800   -2.84271500    0.11211000
C                 2.37443300   -0.46551500    0.01062500
C                 3.79259700   -0.35763300    0.02629900
O                 4.55646400   -1.45254700    0.07973800
C                 4.39082900    0.88952300   -0.01410000
C                 3.58772200    2.03540900   -0.06868000
O                 4.23871200    3.21987500   -0.10653200
C                 2.19578800    1.96620300   -0.08342600
C                 1.60975900    0.70678900   -0.04301800
O                 0.25965800    0.65522000   -0.06001700
C                -1.88319100   -0.32104700   -0.03720800
C                -2.39000600    0.97912900    0.12692300
C                -3.75356000    1.20799900    0.11966900
C                -4.63700700    0.13351800   -0.05679700
O                -5.96233800    0.47004100   -0.04721000
C                -4.14535900   -1.15125900   -0.22454400
C                -2.77327500   -1.38665700   -0.21548800
H                 3.96458300   -2.23926500    0.10830800
H                 5.46956600    0.98204000   -0.00305100
H                 3.59598600    3.94252600   -0.14326100
H                 1.57147000    2.85221300   -0.12446100
H                -1.72892400    1.82562300    0.26498400
H                -6.50440400   -0.32085300   -0.17313200
H                -4.83803800   -1.97753300   -0.36543200
H                -2.40257900   -2.39285800   -0.35045900
O                -0.34643300   -2.91485000    0.08510800
H                 0.39864500   -3.54925800    0.13118700
O                -4.23288300    2.46649700    0.28434600
H                -5.20192300    2.42195100    0.25411100

1 2 2.0 13 1.0 14 1.0
2 3 1.0 29 1.0
3 4 2.0 5 1.5
4
5 6 1.5 12 1.5
6 7 1.5 8 2.0
7 21 1.0
8 9 1.5 22 1.0
9 10 1.0 11 1.5
10 23 1.0
11 12 1.5 24 1.0
12 13 1.0
13
14 15 1.5 20 1.5
15 16 2.0 25 1.0
16 17 1.5 31 1.0
17 18 1.0 19 2.0
18 26 1.0
19 20 1.5 27 1.0
20 28 1.0
21
22
23
24
25
26
27
28
29 30 1.0
30
31 32 1.0
32
```

Figure S6. The minimum of the Cartesian coordinates for quercetin used in this study.

```
C                 2.46911100   -1.14993700   -0.01943400
C                 3.55166100   -1.93754200    0.21216600
C                 4.88673900   -1.36949500    0.33030000
O                 5.84336100   -2.13562500    0.55895500
C                 4.98972700    0.05532000    0.17789000
C                 6.23738200    0.73361500    0.26118400
O                 7.36453200    0.05624500    0.49407100
C                 6.28925100    2.10711100    0.09843200
C                 5.10689300    2.81500500   -0.14688000
O                 5.23183500    4.15250200   -0.29934300
C                 3.86684400    2.18269400   -0.22979100
C                 3.83046300    0.80347200   -0.06417200
O                 2.62039100    0.20385800   -0.15001700
C                 1.07061100   -1.57164000   -0.14263900
C                 0.05049500   -0.61849700   -0.04467500
C                -1.28011300   -0.98537600   -0.14929400
C                -1.64139300   -2.32046800   -0.39318100
O                -2.91397100   -2.74380200   -0.51889200
C                -0.61966900   -3.26809800   -0.49115600
C                 0.71196500   -2.91061700   -0.36323000
H                 7.13738100   -0.89816800    0.58359500
H                 7.23459900    2.63170100    0.15800700
H                 4.36389600    4.54223500   -0.47680700
H                 2.94987500    2.72892200   -0.42279700
H                 0.28503600    0.42454500    0.13753600
H                -3.50974400   -1.99288700   -0.71003600
H                -0.90691900   -4.29753700   -0.67620000
H                 1.47941200   -3.66864200   -0.44829300
O                 3.48262000   -3.27792000    0.36855800
O                -2.23956700   -0.00256000    0.07046000
H                 4.40579200   -3.55192800    0.54734000
C                -3.07356900    0.36630000   -1.00536100
C                -3.38080100    1.85355100   -0.82165100
O                -4.25732600   -0.40154500   -1.02987100
C                -4.29554300    2.06289100    0.37751300
H                -3.89040000    2.21909200   -1.71950400
O                -2.19855700    2.60818800   -0.68495500
C                -5.10358300   -0.26459900    0.12721400
C                -5.52807300    1.19483300    0.23885100
H                -3.76148600    1.76386300    1.29492300
O                -4.72559500    3.40320500    0.46295200
H                -1.66566800    2.14233700   -0.01864100
H                -4.55352700   -0.56269200    1.02972700
C                -6.28417100   -1.21218200   -0.06538500
H                -6.05462300    1.47669800   -0.68842000
O                -6.37870000    1.33605700    1.35720900
H                -3.93329500    3.95996700    0.40108600
H                -5.90332200   -2.20453300   -0.32838600
H                -6.89555700   -0.85425400   -0.90928400
O                -7.02521800   -1.35518700    1.11664700
H                -6.49902900    2.28872900    1.50012800
H                -7.26939900   -0.46481600    1.41671300
H                -2.56882500    0.17201400   -1.95709200

1 2 2.0 13 1.0 14 1.0
2 3 1.0 29 1.0
3 4 2.0 5 1.5
4
5 6 1.5 12 1.5
6 7 1.5 8 2.0
7 21 1.0
8 9 1.5 22 1.0
9 10 1.0 11 1.5
10 23 1.0
11 12 1.5 24 1.0
12 13 1.0
13
14 15 1.5 20 1.5
15 16 2.0 25 1.0
16 17 1.5 30 1.0
17 18 1.0 19 1.5
18 26 1.0
19 20 2.0 27 1.0
20 28 1.0
21
22
23
24
25
26
27
28
29 31 1.0
30 32 1.0
31
32 33 1.0 34 1.0 53 1.0
33 35 1.0 36 1.0 37 1.0
34 38 1.0
35 39 1.0 40 1.0 41 1.0
36
37 42 1.0
38 39 1.0 43 1.0 44 1.0
39 45 1.0 46 1.0
40
41 47 1.0
42
43
44 48 1.0 49 1.0 50 1.0
45
46 51 1.0
47
48
49
50 52 1.0
51
52
53
```

Figure S7. The minimum of the Cartesian coordinates for quercetin-3'-*O*-glucoside used in this study.

```
C                -2.52779600   -0.32381700   -0.05721900
C                -3.15276400   -1.51410400   -0.25423400
C                -4.60947200   -1.60781700   -0.28354700
O                -5.12767200   -2.72523500   -0.47005900
C                -5.33287300   -0.38335300   -0.09311100
C                -6.75510100   -0.33577800   -0.10149800
O                -7.47178400   -1.44687800   -0.28943200
C                -7.40663400    0.87033300    0.08616000
C                -6.65448000    2.03461000    0.28312600
O                -7.35706100    3.17534600    0.45889600
C                -5.26037300    2.02422800    0.29715700
C                -4.61992500    0.80604800    0.10657500
O                -3.26869800    0.81282100    0.12087600
C                -1.08286900   -0.05679900   -0.00637400
C                -0.14484000   -1.08820100   -0.13136700
C                 1.21975900   -0.82162100   -0.07833800
C                 1.65590400    0.49678600    0.10293200
O                 2.99702000    0.81124200    0.24260800
C                 0.72845300    1.52560400    0.21389600
C                -0.63097000    1.26044100    0.16577000
H                -6.85022300   -2.20118000   -0.40640300
H                -8.48840600    0.91638300    0.08112200
H                -6.74953200    3.91777600    0.58730600
H                -4.67640900    2.92535100    0.45012700
H                -0.44819500   -2.11715700   -0.26853400
H                 1.09932500    2.53577400    0.35459200
H                -1.34168300    2.07095500    0.25959400
O                -2.50886700   -2.68475600   -0.43948100
O                 2.07088900   -1.88108500   -0.16493300
H                 2.94953800   -1.60211400   -0.50641600
H                -3.22407200   -3.34434000   -0.55653300
C                 3.87046600    0.56693800   -0.85406000
C                 4.92699700    1.67683600   -0.86482800
O                 4.43574400   -0.72260800   -0.78138300
C                 5.89265900    1.50427500    0.31563200
H                 4.39958600    2.63043800   -0.75389900
O                 5.57971500    1.69606700   -2.10411800
C                 5.39864700   -0.98886700    0.26507000
C                 6.48234800    0.08918900    0.30336000
H                 6.71410600    2.21716000    0.18773600
O                 5.28004300    1.74684500    1.56337000
H                 6.25663000    0.99632800   -2.08496500
H                 5.86180900   -1.92814900   -0.06349500
C                 4.75339000   -1.32041600    1.61753100
H                 7.08141700   -0.04211000    1.21412100
O                 7.31055600    0.01620400   -0.85390200
H                 4.32299200    1.61512000    1.47438400
H                 5.55511800   -1.35590500    2.36360100
H                 4.05242100   -0.54152400    1.92937600
O                 4.15670100   -2.59452400    1.59059900
H                 7.66458900   -0.88076600   -0.93534700
H                 3.23945300   -2.50730300    1.28470400
H                 3.30570900    0.58068700   -1.79121000

1 2 2.0 13 1.0 14 1.0
2 3 1.0 28 1.0
3 4 2.0 5 1.5
4
5 6 1.5 12 1.5
6 7 1.5 8 2.0
7 21 1.0
8 9 1.5 22 1.0
9 10 1.0 11 1.5
10 23 1.0
11 12 1.5 24 1.0
12 13 1.0
13
14 15 1.5 20 1.5
15 16 1.5 25 1.0
16 17 1.5 29 1.0
17 18 1.0 19 1.5
18 32 1.0
19 20 2.0 26 1.0
20 27 1.0
21
22
23
24
25
26
27
28 31 1.0
29 30 1.0
30
31
32 33 1.0 34 1.0 53 1.0
33 35 1.0 36 1.0 37 1.0
34 38 1.0
35 39 1.0 40 1.0 41 1.0
36
37 42 1.0
38 39 1.0 43 1.0 44 1.0
39 45 1.0 46 1.0
40
41 47 1.0
42
43
44 48 1.0 49 1.0 50 1.0
45
46 51 1.0
47
48
49
50 52 1.0
51
52
53
```

Figure S8. The minimum of the Cartesian coordinates for quercetin-4’-*O*-glucoside used in this study.

```
C              -1.17167300    0.84913500   -0.48534200
C              -0.84256500   -0.44685500   -0.74710200
C              -1.79861900   -1.52933600   -0.57454000
O              -1.48519500   -2.71308300   -0.80931500
C              -3.10442700   -1.13974600   -0.09701800
C              -4.14083100   -2.08686800    0.13340400
O              -3.94138200   -3.38719000   -0.08964300
C              -5.37567200   -1.66270000    0.59748200
C              -5.59293700   -0.30259900    0.83786600
O              -6.81886000    0.03810000    1.29147500
C              -4.59919800    0.65357900    0.62327500
C              -3.36976800    0.21048500    0.15814500
O              -2.41855000    1.15838600   -0.04780300
C              -0.32187300    2.04470800   -0.60031800
C              -0.69225500    3.19748500    0.11208700
C               0.07815700    4.34303700    0.04158800
C               1.23394800    4.35101200   -0.75354300
O               1.92324300    5.52888800   -0.75147900
C               1.59809900    3.22043000   -1.46984500
C               0.82685300    2.06540500   -1.39787800
H              -3.01494100   -3.50328500   -0.40593400
H              -6.16711900   -2.38008100    0.77484900
H              -6.86297300    0.99510100    1.42947100
H              -4.76053900    1.71047100    0.80554900
H              -1.57844500    3.21264200    0.73453900
H               2.73093600    5.44222600   -1.27653900
H               2.49159200    3.23937100   -2.08881900
H               1.12595500    1.18688400   -1.95128900
O               0.38838900   -0.81619200   -1.21798900
O              -0.28099300    5.44877600    0.73920500
H               0.38587800    6.13507600    0.57652800
C               1.29759000   -1.29158500   -0.25297900
C               2.03477700   -2.47773000   -0.86251800
H               0.75608800   -1.60766100    0.65487300
O               2.17231800   -0.23190200    0.03785300
C               3.16708400   -2.88029100    0.06483600
H               2.45551800   -2.14087100   -1.82416600
O               1.20678200   -3.59862400   -1.01789100
C               3.19564100   -0.57126800    0.96471600
C               4.04934300   -1.68629400    0.36146200
H               2.72647200   -3.23179400    1.01305300
O               3.97908300   -3.88625700   -0.50064300
H               0.30059200   -3.29977400   -1.23302800
H               2.75936600   -0.92522400    1.91301700
C               3.99459000    0.69757000    1.24237500
H               4.47986500   -1.31863700   -0.58436500
O               5.07320300   -2.03066400    1.27438500
H               3.38498300   -4.60708200   -0.76371900
H               3.29471700    1.49998600    1.49344900
H               4.52168600    0.98960600    0.31889800
O               4.86784400    0.53815800    2.33096400
H               5.46320300   -2.85967600    0.95250100
H               5.38252000   -0.26989200    2.17125800

1 2 2.0 13 1.0 14 1.0
2 3 1.0 29 1.0
3 4 2.0 5 1.5
4
5 6 1.5 12 1.5
6 7 1.5 8 2.0
7 21 1.0
8 9 1.5 22 1.0
9 10 1.0 11 1.5
10 23 1.0
11 12 1.5 24 1.0
12 13 1.0
13
14 15 1.5 20 1.5
15 16 2.0 25 1.0
16 17 1.5 30 1.0
17 18 1.0 19 1.5
18 26 1.0
19 20 1.5 27 1.0
20 28 1.0
21
22
23
24
25
26
27
28
29 32 1.0
30 31 1.0
31
32 33 1.0 34 1.0 35 1.0
33 36 1.0 37 1.0 38 1.0
34
35 39 1.0
36 40 1.0 41 1.0 42 1.0
37
38 43 1.0
39 40 1.0 44 1.0 45 1.0
40 46 1.0 47 1.0
41
42 48 1.0
43
44
45 49 1.0 50 1.0 51 1.0
46
47 52 1.0
48
49
50
51 53 1.0
52
53
```

Figure S9. The minimum of the Cartesian coordinates for quercetin-3-*O*-glucoside used in this study.

```
C                 3.11851500   -0.30832600    0.16806700
C                 2.21240200   -1.30718000    0.32765600
C                 0.77168000   -1.05320200    0.39137000
O                 0.03231800   -2.04541400    0.46150400
C                 0.38259400    0.34492300    0.34795900
C                -0.95488100    0.80891400    0.44134900
O                -1.89107900   -0.12188400    0.75179900
C                -1.27745300    2.13619100    0.26772400
C                -0.24729400    3.06009800    0.01763900
O                -0.62510100    4.34620900   -0.14247100
C                 1.07967600    2.66236000   -0.03579300
C                 1.37746500    1.30903900    0.13107400
O                 2.68344000    0.98299900    0.05854600
C                 4.57852300   -0.41655500    0.06527400
C                 5.32025000    0.71273700   -0.32109400
C                 6.69706600    0.64640600   -0.42838700
C                 7.35842800   -0.55734600   -0.14529300
O                 8.71807000   -0.51375700   -0.28292400
C                 6.63497200   -1.67429800    0.24131200
C                 5.24845800   -1.61252000    0.34879000
H                -2.30280400    2.47371700    0.36225300
H                 0.15493800    4.90146700   -0.28593800
H                 1.88818900    3.36555800   -0.20635600
H                 4.83380900    1.65413600   -0.54404900
H                 9.09993800   -1.37381900   -0.06001600
H                 7.15615500   -2.60244500    0.46314200
H                 4.69675000   -2.49006000    0.65429100
O                 2.54978600   -2.61222000    0.40084600
O                 7.40298300    1.74148700   -0.80633700
H                 8.34222700    1.49746400   -0.82650800
H                 1.68702000   -3.07501700    0.46364700
C                -3.07322300   -0.16608200    0.00543500
C                -3.52566500   -1.61960300    0.06176800
O                -4.02666200    0.69155400    0.58373100
C                -4.86139200   -1.74179000   -0.64188500
H                -3.65228900   -1.87731200    1.12555200
O                -2.61517900   -2.46730400   -0.58160300
C                -5.27250600    0.65806300   -0.10709300
C                -5.84242300   -0.76044500   -0.03086000
H                -4.72262500   -1.48369300   -1.70567800
O                -5.41134400   -3.03472900   -0.51736500
H                -1.74600900   -2.37023100   -0.13995600
H                -5.13297400    0.93223200   -1.16585100
C                -6.18977400    1.68997900    0.54109700
H                -5.98033200   -1.01860900    1.03174300
O                -7.08026300   -0.79498400   -0.71492300
H                -4.71668700   -3.65668100   -0.78647700
H                -5.64754900    2.63657200    0.62155800
H                -6.43351700    1.35553300    1.56221500
O                -7.33661800    1.92695000   -0.23493400
H                -7.32735200   -1.73058400   -0.79322500
H                -7.74241700    1.06400000   -0.41839300
H                -2.86977100    0.13835000   -1.03557800

1 2 2.0 13 1.0 14 1.0
2 3 1.0 28 1.0
3 4 2.0 5 1.0
4
5 6 1.5 12 1.5
6 7 1.0 8 2.0
7 32 1.0
8 9 1.5 21 1.0
9 10 1.0 11 1.5
10 22 1.0
11 12 1.5 23 1.0
12 13 1.0
13
14 15 1.5 20 1.5
15 16 2.0 24 1.0
16 17 1.5 29 1.0
17 18 1.0 19 2.0
18 25 1.0
19 20 1.5 26 1.0
20 27 1.0
21
22
23
24
25
26
27
28 31 1.0
29 30 1.0
30
31
32 33 1.0 34 1.0 53 1.0
33 35 1.0 36 1.0 37 1.0
34 38 1.0
35 39 1.0 40 1.0 41 1.0
36
37 42 1.0
38 39 1.0 43 1.0 44 1.0
39 45 1.0 46 1.0
40
41 47 1.0
42
43
44 48 1.0 49 1.0 50 1.0
45
46 51 1.0
47
48
49
50 52 1.0
51
52
53
```

Figure S10. The minimum of the Cartesian coordinates for quercetin-5-*O*-glucoside used in this study.

```
C                 -3.09607200    0.75229500    0.05854100
C                 -3.31981600    2.05979700    0.36418600
C                 -2.22599600    3.01411500    0.44529100
O                 -2.48387400    4.20086600    0.72849600
C                 -0.90734500    2.50027800    0.18699400
C                  0.24855000    3.32408800    0.25145300
O                  0.14674500    4.62294600    0.54686800
C                  1.49343600    2.77643600   -0.00430300
C                  1.59777500    1.41680100   -0.32313400
O                  2.87194700    0.98137200   -0.55825700
C                  0.49081300    0.57630900   -0.39090100
C                 -0.75354500    1.14715900   -0.13118600
O                 -1.82264100    0.32380100   -0.18926400
C                 -4.07952300   -0.33177600   -0.05432600
C                 -3.65081900   -1.59619900   -0.49342800
C                 -4.54702000   -2.64278400   -0.60519000
C                 -5.89515000   -2.44138200   -0.27508900
O                 -6.69429100   -3.54078600   -0.42071100
C                 -6.32603700   -1.19822500    0.16004200
C                 -5.42778300   -0.14089100    0.27254100
H                 -0.80552400    4.83064600    0.69124700
H                  2.37978400    3.39754100    0.03485100
H                  0.56983000   -0.48388600   -0.58872300
H                 -2.61555400   -1.78073300   -0.75130700
H                 -7.59948100   -3.32868700   -0.15425300
H                 -7.37251600   -1.05046700    0.41576700
H                 -5.77890800    0.82119800    0.61674500
O                 -4.54381400    2.57734600    0.61296400
O                 -4.12271200   -3.85770300   -1.03281100
H                 -4.88651000   -4.45662900   -1.03639100
H                 -4.37610200    3.52540100    0.79285400
C                  3.06279900   -0.25784700   -1.22350100
C                  4.54362300   -0.27207600   -1.60805600
O                  2.72366900   -1.35637400   -0.43561800
C                  5.40171600   -0.43453900   -0.35928300
H                  4.71859900   -1.13006100   -2.26593900
O                  4.91998900    0.87823900   -2.32968500
C                  3.46725600   -1.48906300    0.78000800
C                  4.94246700   -1.64711200    0.42334800
H                  5.28236200    0.45650500    0.27941700
O                  6.75833800   -0.62885300   -0.69511800
H                  4.54878800    1.63624500   -1.84865700
H                  3.33072500   -0.59890000    1.40926800
C                  2.90746000   -2.69929400    1.51923100
H                  5.04883900   -2.53803500   -0.21819700
O                  5.69193000   -1.79930800    1.61200900
H                  6.99715800    0.07488500   -1.31911900
H                  1.81906400   -2.60316300    1.56972700
H                  3.13862700   -3.60879100    0.94174000
O                  3.38531000   -2.76472800    2.83830700
H                  6.62764500   -1.73404100    1.36194600
H                  4.35431400   -2.72197200    2.79671500
H                  2.41336600   -0.29812000   -2.10457000

1 2 2.0 13 1.0 14 1.0
2 3 1.0 28 1.0
3 4 2.0 5 1.5
4
5 6 1.5 12 1.5
6 7 1.5 8 2.0
7 21 1.0
8 9 1.5 22 1.0
9 10 1.0 11 1.5
10 32 1.0
11 12 1.5 23 1.0
12 13 1.0
13
14 15 1.5 20 1.5
15 16 2.0 24 1.0
16 17 1.5 29 1.0
17 18 1.0 19 2.0
18 25 1.0
19 20 1.5 26 1.0
20 27 1.0
21
22
23
24
25
26
27
28 31 1.0
29 30 1.0
30
31
32 33 1.0 34 1.0 53 1.0
33 35 1.0 36 1.0 37 1.0
34 38 1.0
35 39 1.0 40 1.0 41 1.0
36
37 42 1.0
38 39 1.0 43 1.0 44 1.0
39 45 1.0 46 1.0
40
41 47 1.0
42
43
44 48 1.0 49 1.0 50 1.0
45
46 51 1.0
47
48
49
50 52 1.0
51
52
53
```

Figure S11. The minimum of the Cartesian coordinates for quercetin-7-*O*-glucoside used in this study.
